# An Alzheimer’s Disease-Derived Biomarker Signature Identifies Parkinson’s Disease Patients with Dementia

**DOI:** 10.1371/journal.pone.0147319

**Published:** 2016-01-26

**Authors:** Yosef Berlyand, Daniel Weintraub, Sharon X. Xie, Ian A. Mellis, Jimit Doshi, Jacqueline Rick, Jennifer McBride, Christos Davatzikos, Leslie M. Shaw, Howard Hurtig, John Q. Trojanowski, Alice S. Chen-Plotkin

**Affiliations:** 1 Department of Neurology, Perelman School of Medicine at the University of Pennsylvania, Philadelphia, Pennsylvania, United States of America; 2 Department of Psychiatry, Perelman School of Medicine at the University of Pennsylvania, Philadelphia, Pennsylvania, United States of America; 3 Department of Biostatistics and Epidemiology, Perelman School of Medicine at the University of Pennsylvania, Philadelphia, Pennsylvania, United States of America; 4 Department of Radiology, Perelman School of Medicine at the University of Pennsylvania, Philadelphia, Pennsylvania, United States of America; 5 Department of Pathology and Laboratory Medicine, Perelman School of Medicine at the University of Pennsylvania, Philadelphia, Pennsylvania, United States of America; 6 Center for Neurodegenerative Disease Research, Perelman School of Medicine at the University of Pennsylvania, Philadelphia, Pennsylvania, United States of America; Biomedical Research Foundation, UNITED STATES

## Abstract

Biomarkers from multiple modalities have been shown to correlate with cognition in Parkinson’s disease (PD) and in Alzheimer’s disease (AD). However, the relationships of these markers with each other, and the use of multiple markers in concert to predict an outcome of interest, are areas that are much less explored. Our objectives in this study were (1) to evaluate relationships among 17 biomarkers previously reported to associate with cognition in PD or AD and (2) to test performance of a five-biomarker classifier trained to recognize AD in identifying PD with dementia (PDD). To do this, we evaluated a cross-sectional cohort of PD patients (n = 75) across a spectrum of cognitive abilities. All PD participants had 17 baseline biomarkers from clinical, genetic, biochemical, and imaging modalities measured, and correlations among biomarkers were assessed by Spearman’s rho and by hierarchical clustering. We found that internal correlation among all 17 candidate biomarkers was modest, showing a maximum pairwise correlation coefficient of 0.51. However, a five-marker subset panel derived from AD (CSF total tau, CSF phosphorylated tau, CSF amyloid beta 42, *APOE* genotype, and SPARE-AD imaging score) discriminated cognitively normal PD patients vs. PDD patients with 80% accuracy, when employed in a classifier originally trained to recognize AD. Thus, an AD-derived biomarker signature may identify PDD patients with moderately high accuracy, suggesting mechanisms shared with AD in some PDD patients. Based on five measures readily obtained during life, this AD-derived signature may prove useful in identifying PDD patients most likely to respond to AD-based crossover therapies.

## Introduction

Parkinson disease (PD) is the second most common adult-onset neurodegenerative disease, affecting an estimated 1 million people in the United States alone [1,2]. PD is pathologically characterized by the loss of dopaminergic neurons in the substantia nigra of the brain and the presence of Lewy body inclusions containing alpha-synuclein [3].

In addition to the hallmark motor symptoms of bradykinesia, tremor, and rigidity, most PD patients develop cognitive impairment (CI), with up to 83% progressing to dementia [4–7]. PD patients who progress to dementia (PDD) have reduced quality of life and independence [6,8], which adds to the cost of care [9], and results in additional stress for families and caregivers [9].

The need to develop and to understand the role of various biomarkers for PD, and for endophenotypes within PD, is increasingly recognized [10–13]. Defined by the National Institutes of Health Biomarkers Definitions Working Group, a biomarker is “a characteristic that is objectively measured and evaluated as an indicator of normal biological processes, pathogenic processes, or pharmacologic responses to a therapeutic intervention” [14]. While common uses of the term often refer to biochemical or imaging-based measures, in the most generic form, biomarkers might originate from multiple types of data. For the purposes of this paper, we considered clinical, genetic, biochemical, and imaging-based biomarkers.

Although the field is still young, and far from consensus, various candidate biomarkers from these disparate modalities have been reported to associate with cognitive performance in PD (S1 Table). Clinical correlates of poorer cognition in PD include older age [4], greater motor severity [15], male sex [16], a motor phenotype characterized by postural instability and gait disorder (PIGD) [4], increased disease duration [17], and the presence of hallucinations [18] or depression [15]. From the genetics literature, variation in *APOE* [19], *MAPT* [20], *COMT* [20], and *GBA* [21] have been reported to moderate risk for CI in PD. Previously reported biochemical correlates of cognitive decline in PD include lower levels of cerebrospinal fluid (CSF) amyloid beta, specifically the disease-implicated form of amyloid beta known as Aβ42 [22,23], and lower levels of plasma epidermal growth factor (EGF) [24,25], while higher levels of CSF total tau (t-tau) and phosphorylated tau (p-tau) associate with poorer cognition in Alzheimer disease (AD) [23]. Finally, many imaging-based measures have been reported to associate with cognitive performance in PD [26]; as one example, a global pattern of brain atrophy known as the Spatial Pattern of Atrophy for Recognition of AD (SPARE-AD) has been reported to associate with cognitive decline in both AD [27] and PD [28].

It is notable that many of the biochemical and imaging-based biomarkers for cognitive performance in PD are “crossover” biomarkers from AD. No doubt this reflects in part the relative paucity of available candidate PD biomarkers [29]. In addition, however, neuropathological data suggest that CI in PD and in AD may in fact share some mechanistic underpinnings. Specifically, postmortem examinations demonstrate that 29–50% of PDD patients have Aβ plaques and neurofibrillary tangles characteristic of AD [30–34] concurrent with PD-defining alpha-synuclein pathology.

While there is an emerging literature on individual PD biomarkers, how biomarkers from various modalities correlate with each other, and whether they represent the same vs. different underlying biological processes, is not well understood. In the present study, we cross-sectionally evaluated a panel of 17 biomarkers spanning multiple modalities in a densely-characterized cohort of 75 PD patients across a spectrum of cognitive abilities. We sought to understand the relationship of these 17 biomarkers to each other. Additionally, because 5/17 markers in our panel have been strongly implicated in AD, we tested the hypothesis that an AD-derived biomarker signature might identify PDD as well.

## Methods

Please see S1 Methods for additional details.

### Participants

#### UPenn Udall Cohort

The University of Pennsylvania Institutional Review Board approved the protocols and consent procedures. Written informed consent was obtained from all participants in the study. In the case of individuals with limited capacity to consent, consent was obtained from an authorized representative of the patient. Additional details may be found in S1 Methods.

75 patients were prospectively enrolled into the present study from the UPenn subspecialty movement disorders clinic, provided that they met the UK Brain Bank diagnostic criteria for PD [35]. These individuals represent the first 75 subjects prospectively enrolled for the Intensive Assessment Cohort of the UPenn Udall Center, which has a planned enrollment of 150 PD patients. All patients first presented to clinical attention for PD symptoms; however, a subset developed dementia over the course of disease, and these individuals already met criteria for PDD by the time of enrollment into this study. For each patient, 17 candidate biomarkers (Table 1, S1 Table) were captured within one year, representing the baseline visit for each patient’s enrollment into the study.

**Table 1:** Comparison of biomarker data among PD-CN, PD-MCI, and PDD patients.

Biomarker Modality	Features	PD-CN	PD-MCI	PDD	P-value
**Clinical**	**Sex**				0.347^a^
** **	*Male (%)*	35 (74%)	15 (75%)	8 (100%)	
** **	*Female (%)*	12 (26%)	5 (25%)	0 (0%)	
** **	**Age at Plasma Median in yrs (IQR)**	66 (61.5–71.0)	66 (63.0–70.3)	77.5 (71.8–79.0)	**0.003**^b^
** **	**Disease Duration Median in yrs (IQR)**	6.7 (4.7–11.3)	6.9 (3.9–10.9)	10.5 (5.4–14.0)	0.696^b^
** **	**UPDRS III Median (IQR)**	19 (13.3–25.0)	23 (18.0–32.5)	34.5 (30.0–41.5)	**<0.001**^b^
** **	**MODHY Median (IQR)**	2.0 (2.0–2.5)	2.5 (2.0–3.0)	2.75 (2.0–3.0)	**0.009**^b^
** **	**GDS Median (IQR)**	2.0 (1.0–4.0)	3.0 (2.0–6.0)	3.0 (1.75–3.0)	0.179^b^
** **	**Tremor:PIGD Ratio Median (IQR)**	0.8 (0.0–1.5)	0.5 (0.1–1.7)	0 (0–1.4)	0.676^b^
** **	**UPDRS Thought Disorder Median (IQR)**	0.0 (0.0–1.0)	1.0 (0.0–1.0)	0.0 (0.0–1.0)	0.51^b^
**Genetic**	***APOE* E4 Allele Count**				0.534^a^
** **	*0 (%)*	33 (70%)	14 (70%)	4 (50%)	
** **	*1 (%)*	14 (30%)	6 (30%)	4 (50%)	
** **	*2 (%)*	0 (0%)	0 (0%)	0 (0%)	
** **	***MAPT* H1/H1**				0.592^a^
** **	*No (%)*	15 (32%)	5 (25%)	1 (13%)	
** **	*Yes (%)*	32 (68%)	15 (75%)	7 (88%)	
** **	***GBA* Mutant**				1^a^
** **	*No (%)*	45 (96%)	19 (95%)	8 (100%)	
** **	*Yes (%)*	2 (4%)	1 (5%)	0 (0%)	
** **	***COMT* Met Allele Count**				0.774^a^
** **	*0 (%)*	14 (30%)	6 (30%)	2 (25%)	
** **	*1 (%)*	21 (45%)	11 (55%)	3 (38%)	
** **	*2 (%)*	12 (26%)	3 (15%)	3 (38%)	
**Biochemical**	**CSF Aβ42 Median in pg/mL (IQR)**	278.0 (232.0–303.7)	253.5 (210.5–281.0)	169.8 (149.4–217.3)	**0.014**^b^
** **	**CSF t-tau Median in pg/mL (IQR)**	40 (33.5–50.0)	44 (35.5–64.3)	57.2 (20.2–97.5)	0.637^b^
** **	**CSF p-tau Median in pg/mL (IQR)**	20 (14.0–25)	18 (15.75–28.5)	22.4 (14.8–24.6)	0.718^b^
** **	**Plasma EGF Median in pg/mL (IQR)**	17.7 (10.6–52.1)	30 (13.4–76.74)	91 (62.6–196.4)	**0.013**^b^
**Imaging**	**SPARE-AD Median (IQR)**	-0.62 (-1.24–0.39)	-0.15 (-0.68–0.23)	0.64 (0.39–0.82)	**0.011**^b^

a. Fisher-exact test

b. Kruskal-Wallis non-parametric one-way ANOVA

**Bold** indicates an uncorrected p-value <0.05.

Candidate biomarkers were nominated from the existing literature on cognitive biomarkers in PD or AD, and they spanned multiple types of data: clinical, genetic, biochemical, and imaging [36,37].

#### ADNI Cohort

Background information on the Alzheimer’s Disease Neuroimaging Initiative (ADNI) cohort was recently reviewed [36], and appears in the ADNI database (adni.loni.usc.edu) and the S1 Methods.

For the current study, CSF Aβ42, CSF t-tau, CSF p-tau, SPARE-AD score, and *APOE* genotype were downloaded from the ADNI website for all cognitively normal (n = 109) and AD (n = 101) patients with complete information available for all five biomarkers.

### Neuropsychological Assessment

The Mattis Dementia Rating Scale-2 (DRS) was used to assess cognitive performance [38]. Age-adjusted scores were used for all analyses. The 15-item Geriatric Depression Scale was used to assess presence of depressive symptoms as previously described [39]. The presence and severity of hallucinations was ascertained by the Thought Disorder item of the Unified PD Rating Scale (UPDRS) Part I [40]. The Hopkins Verbal Learning Test-Revised (HVLT-R) [41,42] was used as a second test of memory domain function, as previously described [43]. HVLT-R scores for total immediate free recall and recognition discrimination [43], standardized from published norms [42], were analyzed. For those patients for whom severity of dementia prevented completion of the HVLT-R, a floor score of 20 (T-score of 20 = three standard deviations below the mean) was used.

### Classification of PD patients as PD-CN, PD-MCI, or PDD

Cognitive status of our PD cohort, *i*.*e*. cognitively normal (PD-CN), mild cognitive impairment (PD-MCI), or dementia (PDD) was determined by expert clinical consensus at the UPenn Udall Center as previously described [44]. In brief, data considered for assignment of cognitive category included treating clinician impression, clinical chart data, psychometric test data, and measures of function in activities of daily living. Additional details are provided in S1 Methods.

### Motor Assessment

Motor severity of PD symptoms was assessed by the UPDRS Part III (UPDRS-III) score [40] and by the Modified Hoehn and Yahr (MODHY) score [45,46].

### Genetic Testing

Peripheral blood DNA was genotyped for *MAPT*, *COMT*, and *APOE* variants using real-time allelic discrimination with Applied Biosystem (ABI) TaqMan probes as previously described [19].

*GBA* mutation analysis was performed as previously described [47] using long-range PCR followed by sequencing of all 11 *GBA* exons and intron-exon boundaries.

Additional details are provided in S1 Methods.

### Biochemical Testing

CSF Aβ42, t-tau, and p-tau were measured as previously described, using the Innogenetics (INNOBIA AlzBio3) reagent on the xMAP Luminex platform [23,48].

Plasma EGF was measured as previously described, using a commercially available enzyme-linked immunosorbent assay (R&D Systems) [25].

Additional details of sample collection, measurement, and quality control are provided in S1 Methods.

### Imaging

A SPARE-AD score was assigned for each participant as previously described [28]. In brief, the SPARE-AD score reflects the overall similarity between pattern of atrophy seen in a particular individual and a generic pattern reflective of AD.

### Statistical Analysis

All statistical analyses were performed in R (http://www.r-project.org). Additional details are provided in the S1 Methods, and scripts are available in the S1 Scripts.

#### Multiple imputation

A panel of 17 biomarkers was assessed in all patients for a 98% complete dataset (1251/1275 data points available). The 24 missing data points were multiply imputed using the “mi” package in R [49].

#### Bivariate analyses

Bivariate comparisons of PD-CN vs. PDD for candidate biomarkers were evaluated by the non-parametric Mann-Whitney U-test, with Bonferroni correction.

#### Spearman correlation, hierarchical clustering, and logistic regression classifier

Pairwise Spearman correlation coefficients were calculated for assessment of internal correlations among continuous markers and markers with at least 5 categories, comprising 12 candidate markers. Partial pairwise Spearman correlation coefficients, adjusted for cognition (age-adjusted DRS score), were similarly calculated.

Patients and biomarkers were hierarchically clustered by Euclidean distance using average linkage, and heatmaps generated for visualization. Prior to clustering, distributions for biomarkers were tested for normality (Shapiro-Wilk test); biomarkers that were non-normally distributed were log-transformed (CSF t-tau, age, disease duration, plasma EGF), then standardized by setting the mean of each variable to zero with a standard deviation of one.

For classification of AD vs. normal control samples, a five-marker logistic regression classifier was trained on ADNI data and ten-fold cross-validated. The classifier was then evaluated for performance in identifying PDD patients in the UPenn Udall cohort.

## Results

### Patient characteristics

**Fig 1:**
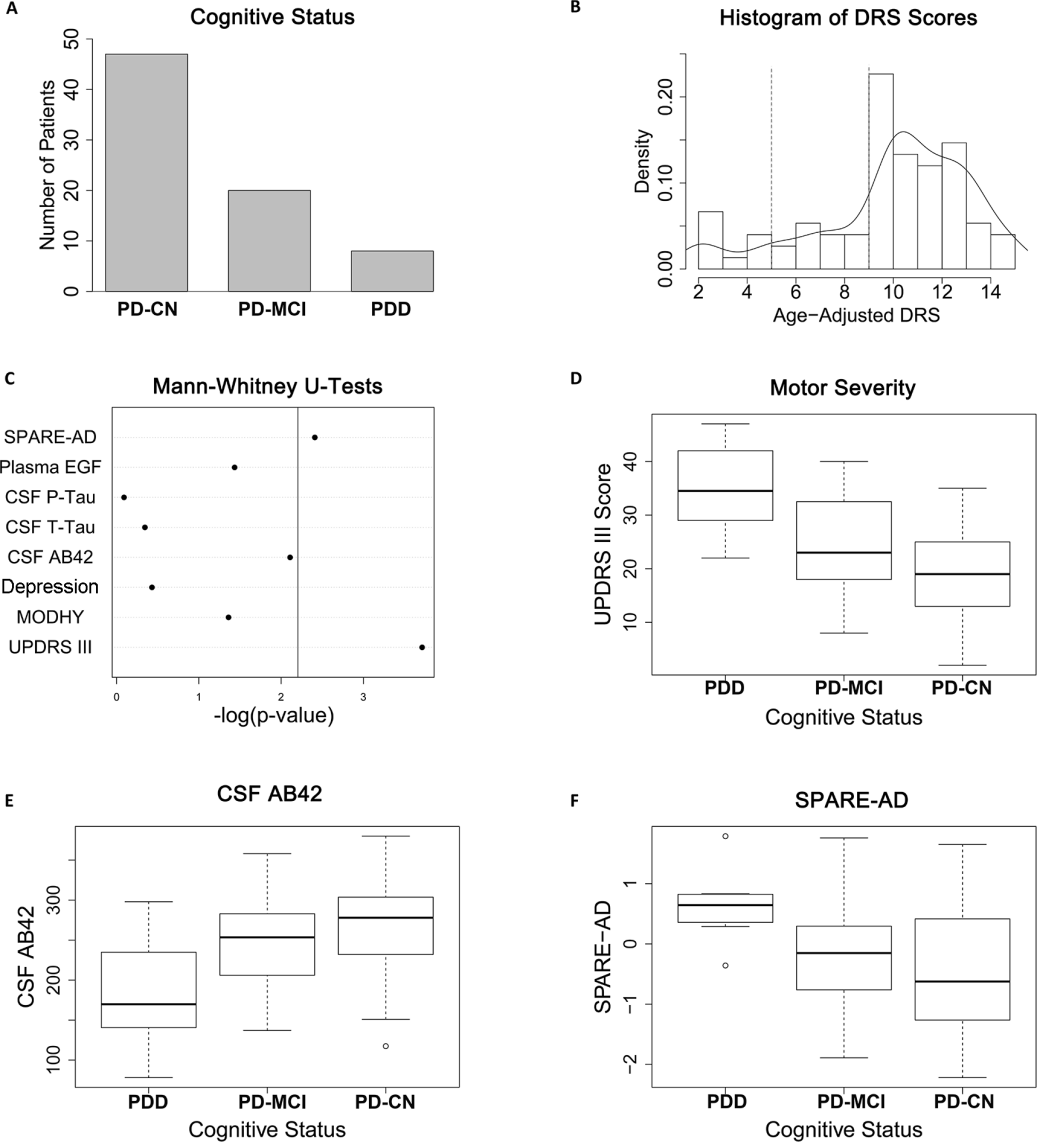
Cohort characteristics and bivariate cross-sectional analyses. **(A)** By consensus clinical determination, 47/75 patients were classified as PD-CN, 20/75 PD-MCI, and 8/75 as PDD. **(B)** Histogram and kernel density plot of age-adjusted DRS scores for the study cohort. Dashed vertical lines represent separations between PDD vs. PD-MCI vs. PD-CN ranges for DRS performance. **(C)** A bivariate analysis of eight candidate markers previously reported to associate cross-sectionally with cognition confirms associations between PDD and two candidate markers in the present study: Unified Parkinson’s Disease Rating Scale Motor score (UPDRS III, corrected p = 0.002) and Spatial Pattern of Atrophy for Recognition of AD score (SPARE-AD, corrected p = 0.031). An additional marker–CSF measures of Aβ42 –trended towards association with PDD (corrected p = 0.062). Vertical line indicates corrected p<0.05. EGF = Epidermal Growth Factor. MODHY = Modified Hoehn and Yahr. The other nine candidate markers assessed in this study have previously been reported to associate with longitudinal decline in cognition. **(D)–(F)** Boxplots of the distribution of UPDRS-III score, CSF Aβ42 levels, and SPARE-AD score among the three cognitive classes. Median and interquartile range are shown.

Seventy-five PD patients were prospectively enrolled at the UPenn Udall Center. Of these 75 patients, 47 (63%) were classified as PD-CN, 20 (27%) as PD-MCI, and 8 (11%) as PDD (Table 1, Fig 1A). Objective assessment with the DRS corroborated a range of cognitive performance (Fig 1B).

### Bivariate cross-sectional analysis of candidate biomarkers demonstrates an association between cognition and two candidate biomarkers

We first sought to replicate previously-reported findings [15,23,25,28] of differences between PD-CN and PDD patients in our current cohort. Of 17 candidate biomarkers, eight (UPDRS-III, MODHY, depression, CSF Aβ42_,_ CSF t-tau, CSF p-tau, plasma EGF, SPARE-AD) had been previously shown to associate cross-sectionally (vs. longitudinally) with baseline cognition in either PD or AD [15,23,25,28].

After Bonferroni correction, significant associations were detected between PDD and two candidate biomarkers. Specifically, PDD patients had greater motor severity as measured by the UPDRS-III (corrected p = 0.002) and exhibited a global brain atrophy pattern similar to AD as captured by the SPARE-AD score [28] (corrected p = 0.031). In addition, PDD patients trended towards lower levels of CSF Aβ42 (corrected p = 0.062). PD-MCI patients had values intermediate to PD-CN and PDD for these three biomarkers (Table 1, Fig 1C–1F).

### 17 candidate biomarkers do not demonstrate substantial internal correlation

**Fig 2:**
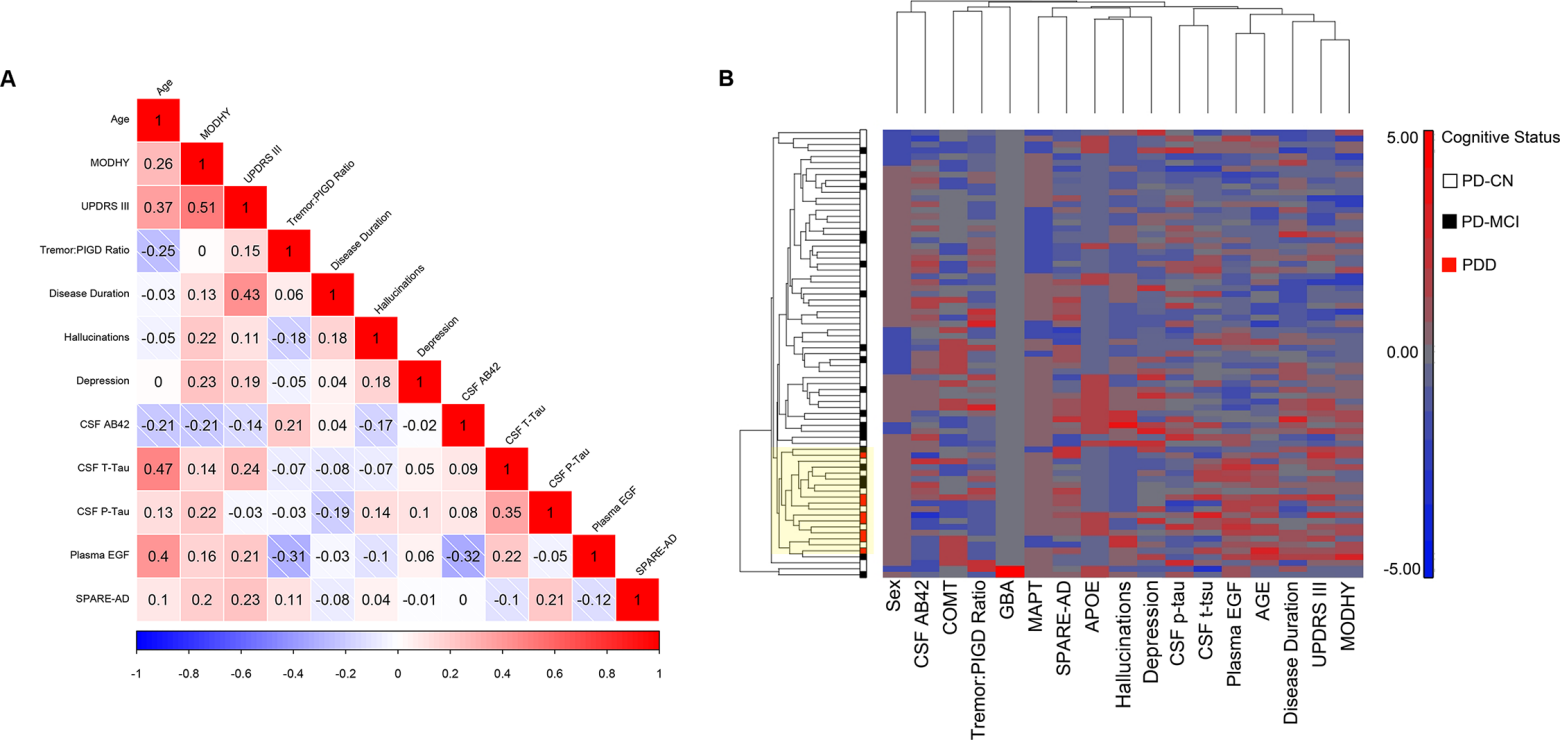
Correlations among candidate biomarkers for cognition. **(A)** Pairwise Spearman correlation coefficients were calculated for the candidate biomarkers for cognition in PD across the entire cohort. With a few exceptions (*e*.*g*. MODHY and UPDRS-III scores (ρ = 0.51), candidate biomarkers did not show high correlations. Shades of red indicate a positive correlation coefficient, white indicates a correlation coefficient of zero, and shades of blue indicate a negative correlation coefficient. The correlation coefficient for each pairwise comparison is reported in the corresponding box. Only 12 candidate biomarkers are shown because five markers are categorical variables with relatively few categories. **(B)** Hierarchical clustering of biomarker candidates does not suggest a high degree of internal correlation among the 17 markers assessed. Both patients and biomarkers were clustered by Euclidean distance using average linkage, with the patient dendrogram shown to the left of the heatmap and the biomarker dendrogram shown above the heatmap. Each column represents one of 17 biomarkers, and each row represents a patient, with PD-CN (white), PD-MCI (black), and PDD (red) individuals indicated by color. On the heatmap, darker red indicates higher marker levels, and darker blue indicates lower marker levels relative to the mean. A branch that captured all the PDD subjects is highlighted in yellow.

We next sought to understand the internal relationships of the 17 candidate biomarkers. As expected, the two most closely correlated markers in our 75-subject cohort were MODHY and UPDRS-III score, with a Spearman correlation coefficient of 0.51 (Fig 2A). On the whole, however, we did not find that our 17 candidate biomarkers were highly internally correlated, in analyses performed with (S1 Fig) or without (Fig 2A, S2 Table) adjustment for cognition, suggesting that they may be capturing different biological signals.

We performed hierarchical cluster analyses to further explore correlations among candidate biomarkers, as well as among individuals in the cohort. As shown in Fig 2B, this cluster analysis corroborated our finding that candidate biomarkers were not highly internally correlated. Examining the patient dendrogram, however, we observed that at a high branchpoint, one cluster captured all the PDD subjects, with PD-CN and PD-MCI individuals admixed.

### A subset of five AD-derived biomarkers is highly correlated in AD but not in PD

In AD, five of the 17 candidate biomarkers assessed here have been reported to demonstrate substantial classification utility: CSF Aβ42, CSF t-tau, CSF p-tau, the SPARE-AD pattern of global brain atrophy, and genotype at the *APOE* locus [19,22,23,28]. We therefore investigated these five AD-derived markers in 109 cognitively normal and 101 AD subjects from the ADNI cohort.

**Fig 3:**
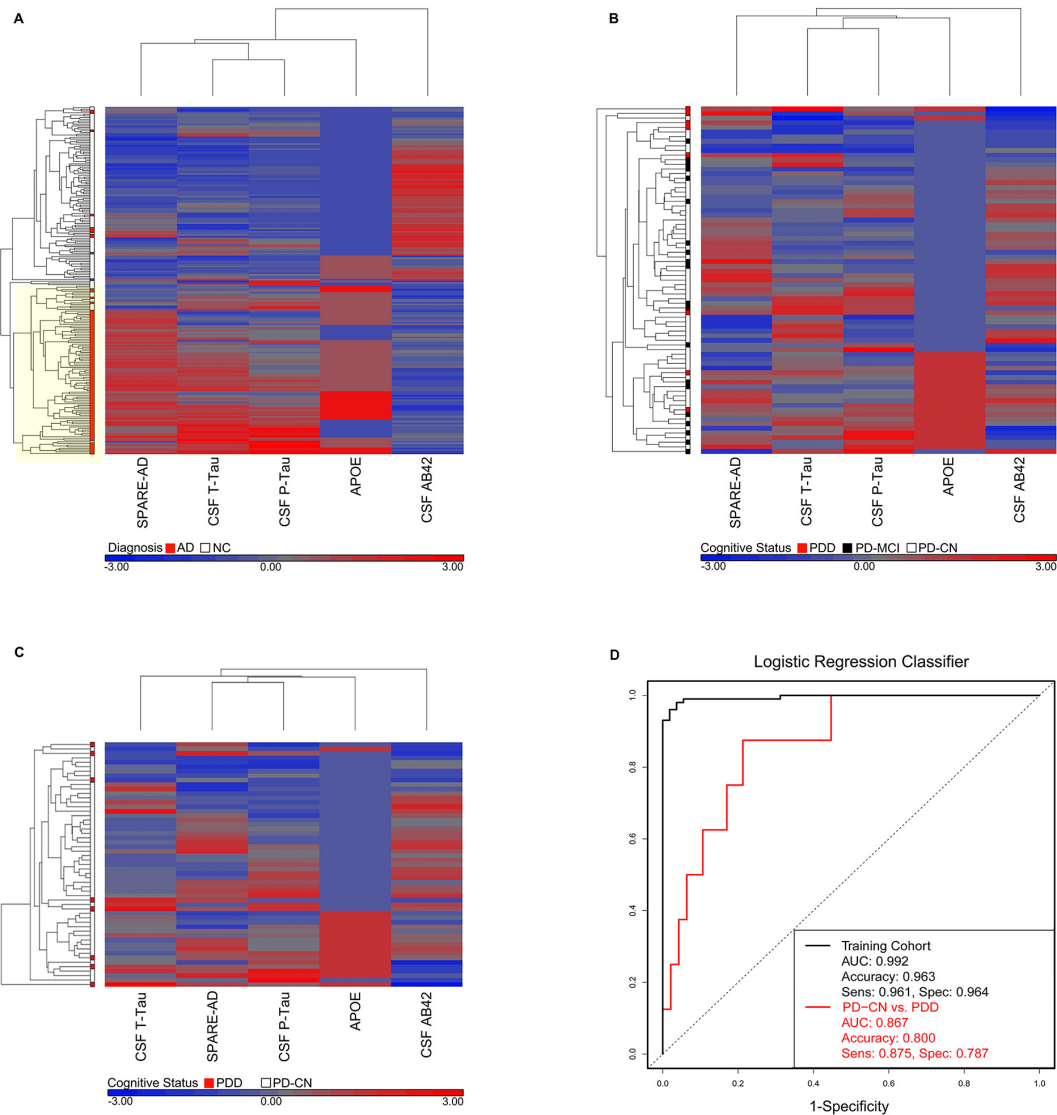
An Alzheimer’s Disease-derived classifier for PDD. **(A)** Hierarchical clustering of five AD-derived biomarkers (CSF Aβ42, CSF t-tau, CSF p-tau, SPARE-AD score, and *APOE* genotype) using data from AD and normal controls (NC) in the ADNI cohort. In AD, these five markers are highly correlated with each other. Moreover, clustering of individuals using these five markers produces a branch highly enriched in AD (yellow highlight). **(B)** Hierarchical clustering of the same five biomarkers using data from the full UPenn Udall cohort (n = 75). In PD, these five markers demonstrate less internal correlation. **(C)** Hierarchical clustering of the five AD-derived biomarkers using data from only PD-CN and PDD patients (n = 55). Even when only the extreme ends of the PD cognitive spectrum are included, less internal correlation is seen among these five markers in PD than in AD. (**D)** A logistic regression classifier (black curve) using the five AD-derived biomarkers discriminates AD from NC in the ADNI cohort with high accuracy. Accuracy, area under the curve, sensitivity, and specificity were assessed by ten-fold cross-validation, using the training cohort of ADNI subjects. Applying the exact same AD-derived classifier to the UPenn Udall cohort discriminates PD-CN from PDD patients with 80% accuracy as well (red curve).

CSF Aβ42, CSF t-tau, CSF p-tau, SPARE-AD score, and *APOE* genotype were strongly correlated in ADNI subjects (Fig 3A). Moreover, evaluating subjects by hierarchical cluster analysis based on these five markers in the ADNI cohort resulted in a large cluster highly enriched in AD patients.

When we performed a similar analysis using the five AD biomarkers in the UPenn Udall cohort, however, we found that they were less internally correlated in PD patients (Fig 3B). Omitting the PD-MCI group, in order to focus on the extremes of PDD and PD-CN, did not change this result (Fig 3C).

### An AD-derived classifier identifies PDD subjects with 80% accuracy

Although the five-marker panel of CSF p-tau, CSF t-tau, SPARE-AD score, *APOE* genotype, and CSF Aβ42 shows less internal correlation in the PD cohort than in ADNI, these five markers might nevertheless prove useful in classifying PD-CN vs. PDD patients. Indeed, some have argued that in at least some PD patients, dementia results from an “Alzheimer”-ization of the brain concomitant with the development of Lewy body inclusions containing alpha-synuclein [50].

To evaluate this hypothesis, we first trained a logistic regression classifier using these five markers to classify cognitively normal vs. AD subjects from ADNI.

As expected based on the cluster analyses, a classifier using CSF p-tau, CSF t-tau, SPARE-AD score, *APOE* genotype and CSF Aβ42 measures performed well in discriminating AD from cognitively normal ADNI subjects. Indeed, in ten-fold cross-validation within the ADNI cohort, our logistic regression classifier separated AD vs. normal subjects with an accuracy of >96% (95% CI 0.93–0.98, area under the receiver operating curve (AUC) 0.99, sensitivity 0.96, specificity 0.96, Fig 3D).

The logistic regression classifier was trained in the ADNI cohort to identify AD; we next asked if this exact classifier could discriminate PD-CN vs. PDD patients from the UPenn Udall cohort. Indeed, the five-marker AD-derived classifier distinguished PD-CN from PDD subjects with 80% accuracy (95% CI 0.67–0.90, AUC 0.87, sensitivity 0.88, specificity 0.79, Fig 3D). Re-running this analysis using the 74 PD individuals with no imputed data (one SPARE-AD score for one PD patient was imputed) did not change the results (accuracy 0.80, AUC 0.86).

**Fig 4:**
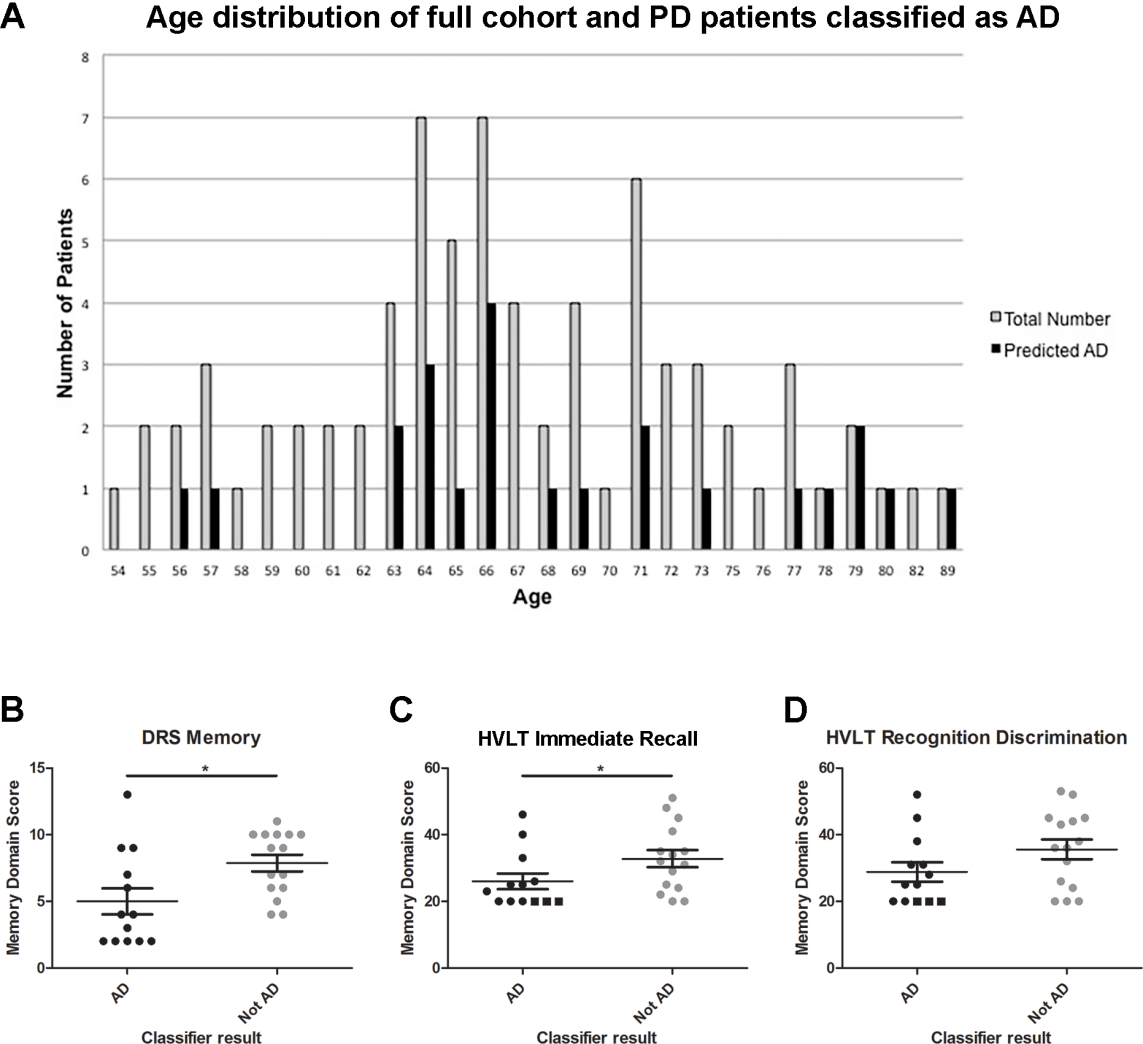
Age and memory impairment in PD patients classified as AD-like. **(A)** Histogram depicting age frequencies for all 75 PD patients in the study (grey bars) vs. subset of PD patients classified as having an AD-like biomarker profile (black bars). AD-like individuals represent a range of ages. **(B)** DRS-memory domain scores for cognitively impaired (PDD and PD-MCI) PD patients classified as AD-like (black) vs. not AD-like (grey). Age-adjusted DRS memory domain scores are shown. *Two-tailed Mann-Whitney p <0.05. **(C-D)** Standardized scores (T-scores, where 50 is the mean and each 10-point change from 50 represents one standard deviation) for total immediate free recall **(C)** and recognition discrimination **(D)** from the Hopkins Verbal Learning Test-Revised (HVLT-R) for cognitively impaired (PDD and PD-MCI) PD patients classified as AD-like (black) vs. not AD-like (grey). Of note, 3/13 PD patients classified as AD-like were unable to complete testing due to severity of dementia; these individuals are denoted by a square symbol and assigned a T-score of 20. All PD patients classified as not AD-like were able to complete testing. *Two-tailed Mann-Whitney p <0.05.

Because our cohort was prospectively enrolled, PD patients in different cognitive categories were not age-matched, reflecting, instead, the innate distribution of ages found in PD patients across a spectrum of cognitive performance. As a consequence, PDD patients were significantly older than individuals in other cognitive categories. We therefore asked whether the older age of the PDD individuals could be driving the ability of our AD-derived classifier to identify them. As shown in Fig 4A, however, PD patients classified as AD-like by this five-marker classifier represent a range of ages, with two-thirds falling within the interquartile range of ages for the PD-CN group.

We further asked whether each of the markers individually associated with cognitive category and whether this association persisted after adjustment for age. Of the five markers comprising our AD-derived classifier, only two associated significantly with cognitive category in bivariate logistic regressions–SPARE-AD score and CSF Aβ42. Notably, in the five-marker AD-derived classifier, SPARE-AD score contributed most to classification, with the largest coefficient. SPARE-AD score remained significantly associated with cognitive category after adjustment for age, while the p-value for association of CSF Aβ42 levels with cognitive category increased from 0.005 to 0.088 after adjustment for age. In both cases, the addition of age as a covariate did not change the direction and minimally affected the coefficient for the biomarker of interest. Moreover, in both cases, the model containing both age and the biomarker of interest was more informative, as indicated by a lower Akaike Information Criterion value, than a model in which cognitive category was predicted from age alone (S3 Table).

### The memory domain is impaired in PD-MCI and PDD patients with an AD-like biomarker profile

Having demonstrated that our AD-derived classifier is not simply selecting individuals with an aged biomarker profile, we hypothesized that the PD patients deemed more AD-like might exhibit memory impairment similar to that seen in AD. Among PDD and PD-MCI patients in our cohort, 13 were classified by the five-marker panel as AD-like, whereas 15 were not. These two groups were compared for performance on memory-specific tests. We note that PD-MCI individuals were also included in this analysis because nearly all the PDD patients were classified as AD-like, with the consequence that an analysis confined to PDD individuals would not allow comparison between balanced groups with vs. without an AD-like biomarker profile.

On the memory domain portion of the DRS, the 13 PD patients identified as AD-like demonstrated significantly poorer performance, compared to the 15 PD individuals deemed not AD-like by our logistic regression classifier (two-tailed Mann-Whitney p-value = 0.012, Fig 4B). Similar results were observed for a second test of memory, the Hopkins Verbal Learning Test-Revised (HVLT-R) [41,42], for which average T-scores (standardized from published norms [42]) on immediate free recall (Fig 4C) and recognition discrimination (Fig 4D) were lower in AD-like PD individuals, compared to PD individuals classified as not AD-like. While differences in total immediate recall were significant between groups (two-tailed Mann-Whitney p-value = 0.050), differences in recognition discrimination showed only a non-significant trend (two-tailed Mann-Whitney p-value = 0.129).

## Discussion

Biomarker studies in neurodegenerative diseases are becoming increasingly common. However, in most cases, a small number of candidate markers, usually from the same modality, are used to predict an outcome of interest. As a consequence, we gain an increasing number of associations between individual markers and various clinical outcomes, but the relationships among these individual markers remain unclear.

In PD, the clinical outcome of CI and dementia has been studied in detail, with the advent of multiple candidate biomarkers reported to correlate with current cognitive state and future cognitive decline [4,15–21,25,51]. The focus on CI and dementia in PD reflects the fact that this aspect of PD occurs in the vast majority of patients [4–7], exacting a significant personal and financial cost [9]. Moreover, while there are currently no neuroprotective therapies and only marginally effective symptomatic therapies for PD-MCI and PDD, at least two broad mechanisms for explaining this PD phenotype exist.

Specifically, Braak and Braak have hypothesized that dementia may develop in PD as alpha-synuclein pathology spreads from brainstem-limited patterns to involvement of the neocortex [52,53]. An alternative, but not mutually exclusive, mechanistic hypothesis for the development of dementia in PD has emphasized the role of the concomitant AD-defining senile plaques and neurofibrillary tangles found in a large proportion of PD patients; put simply, proponents of this theory posit that an AD-like process may drive the development of PDD in at least some patients [34,50,54–57].

Here, we first sought to understand relationships among multiple individual markers that have been linked to CI and dementia in PD. To this end, we evaluated a deeply characterized cohort of PD patients, ascertaining their characteristics across 17 candidate biomarkers spanning multiple modalities. These markers have not previously been studied together in a cohort of patients.

Somewhat surprisingly, these 17 markers did not show a high degree of internal correlation, with the two most highly-correlated markers, MODHY and UPDRS-III score, demonstrating a correlation coefficient of just 0.51 across the 75 PD patients in our cohort.

Our second objective in this study was to evaluate the hypothesis that an AD-like process may drive the development of PDD. To address this question, we evaluated a subset of five markers strongly linked to AD–CSF p-tau, CSF t-tau, SPARE-AD score, *APOE* genotype, and CSF Aβ42 –in our PD patients. Intriguingly, a logistic regression classifier using these five markers trained to recognize AD could also discriminate PD-CN from PDD subjects. Indeed, classification accuracy was high, at 80%, with balanced sensitivity and specificity, despite the relative lack of internal correlation among these markers in PD subjects compared to AD subjects. Our finding is thus consistent with the hypothesis that the development of dementia in PD and AD may share biological mechanisms, manifesting as a shared biomarker signature. Supporting this hypothesis is our clinical finding that cognitively impaired PD patients deemed AD-like have poorer performance on both the memory subsection of the Mattis DRS and on the HVLT-R recall than cognitively impaired PD patients who are classified as not AD-like. We note here that AD-like PD patients had lower scores for both immediate free recall and recognition on the HVLT-R, but only the former difference was statistically significant. It is possible that this reflects small sample numbers and that a larger sample size would show differences between groups on both aspects of the HVLT-R. However, some PD patients have been reported to show impairment of free recall with relative sparing of recognition memory compared to AD patients [43,58,59], raising the possibility that even in cognitively impaired PD patients with an AD-like biomarker profile, the specific nature of the memory deficit may still differ from AD.

Our *in vivo* biomarker findings corroborate multiple lines of evidence suggesting some degree of shared pathophysiology in AD and PDD. First, postmortem neuropathological examination reveals that up to 50% of PDD patients have concomitant AD pathology [30–34]. Second, biochemical investigations suggest that the individual proteins aggregating in PD and in AD–and in particular the alpha-synuclein protein characteristic of PD, and tau protein characteristic of AD–may each enhance the formation of pathological inclusions of the other protein [60–63].

Several limitations of our study should be acknowledged. First, we studied a relatively small sample of PD patients. Because of this limitation, we chose not to develop and cross-validate classifiers within the PD group itself at the present time, as our numbers within this group might be insufficient to develop robust classifiers. In addition, further validation in larger cohorts of the predictive ability of our five-marker AD-derived classifier to correctly identify PDD patients would be a valuable addition to the current report. It is important to note, however, that the strength of our study lies not in the number of subjects enrolled, but in the depth of their characterization across multiple modalities, resulting in a dense and highly complete dataset collected from a prospectively enrolled cohort, which will be made publicly available. Indeed, it is precisely the depth of characterization in our cohort that allows us to demonstrate, for the first time, the relative lack of correlation among these disparate cognitive biomarkers.

Second, our cohort has moderate PD, with a median disease duration of 6.9 years. Thus, it remains to be seen whether our findings, and in particular the ability of an AD-derived classifier to correctly identify PDD subjects, would generalize to other stages of disease.

Third, as is frequently encountered in the clinic, the PDD patients in our study are older than the PD-MCI or PD-CN patients. We guarded against the possibility that our AD-derived classifier may simply be detecting an aged biomarker profile by evaluating the age distribution of individuals deemed AD-like and by performing further analyses adjusting for age. However, it is possible that at least some of the signal detected by our classifier could still be due to increased age. In this regard, given the younger age of the PD-MCI individuals in our cohort, it will be interesting to see if those classified as AD-like now will be more likely to develop dementia in the future.

In summary, we show that it is possible to use a small panel of biomarkers to discriminate PD patients with and without dementia, and, further, that the discriminating biomarkers may reflect an underlying process shared with AD. We note that the concept of shared pathophysiology between AD and PD/PDD has important ramifications. In AD, therapies targeting the generation and deposition of Aβ are in clinical trials now [64–68]. Should any of these agents show promise in large human AD trials, evidence for shared pathophysiology in these two diseases might suggest the evaluation of these therapeutics to prevent dementia in PD-CN subjects at greatest risk of developing dementia, or to treat manifest dementia in PDD patients. Moreover, the development of classifiers selecting the most AD-like of PD patients–and thus the PD patients most likely to respond to “crossover” therapies–may be of substantial practical use.

## Supporting Information

S1 Dataset Biomarker data for Parkinson’s Disease patients. (CSV)

S2 Dataset Biomarker data for ADNI subjects. (CSV)

S1 Fig Partial correlations among candidate biomarkers, adjusted for cognitive performance. Pairwise partial Spearman correlation coefficients were calculated for the candidate biomarkers for cognition in PD across the entire cohort, using the age-adjusted DRS score to control for cognitive performance. Candidate biomarkers did not show high correlations. Shades of red indicate a positive correlation coefficient, white indicates a correlation coefficient of zero, and shades of blue indicate a negative correlation coefficient. The correlation coefficient for each pairwise comparison is reported in the corresponding box. Only 12 candidate biomarkers are shown because five markers are categorical variables with relatively few categories.
(DOCX)

S1 Methods (DOCX)

S1 Scripts (DOCX)

S1 Table Previously-reported associations between candidate biomarkers and cognition. 16 markers previously reported in the literature are summarized, but 17 were evaluated in the present study, since motor severity was assessed by both UPDRS-III and MODHY. Full references are provided in the main text.
(DOCX)

S2 Table R^2^ values for correlation of categorical markers. We assessed the degree of internal correlation among our candidate markers by calculating R^2^ values for logistic and linear regressions involving one or more categorical markers. For categorical-categorical biomarker comparisons (shaded in grey), logistic regressions were performed and the McFadden R^2^ value is reported. For categorical-continous biomarker comparisons, linear regressions were performed after inspection of data for normality, and log-transformation of non-normal biomarkers. The degree of correlation observed among markers was low.
(DOCX)

S3 Table Effect of age on classifier. Logistic regression models predicting cognitive category (PDD vs. PD-CN) based on indicated variables. Five models are shown. In each case, the addition of the biochemical or imaging biomarker improves the model compared to age alone, as reflected by a lower Akaike Information Criterion (AIC) measure. In each case, the addition of age as a covariate does not change the direction and minimally changes the magnitude of effect for the biochemical or imaging biomarker, as reflected by the coefficient.
(DOCX)
