# Supplementary material for: An Alzheimer’s Disease-Derived Biomarker Signature Identifies Parkinson’s Disease Patients with Dementia
Source: PLoS One. 2016 Jan 26;11(1):e0147319. doi: 10.1371/journal.pone.0147319 (PMC4727929; doi:10.1371/journal.pone.0147319)
Supplement: S1 Fig — Pairwise partial Spearman correlation coefficients were calculated for the candidate biomarkers for cognition in PD across the entire cohort, using the age-adjusted DRS score to control for cognitive performance. Candidate biomarkers did not show high correlations. Shades of red indicate a positive correlation coefficient, white indicates a correlation coefficient of zero, and shades of blue indicate a negative correlation coefficient. The correlation coefficient for each pairwise comparison is reported in the corresponding box. Only 12 candidate biomarkers are shown because five markers are categorical variables with relatively few categories. (DOCX) [file pone.0147319.s003.docx]

**S1 Fig. Partial correlations among candidate biomarkers, adjusted for cognitive performance.**

Pairwise partial Spearman correlation coefficients were calculated for the candidate biomarkers for cognition in PD across the entire cohort, using the age-adjusted DRS score to control for cognitive performance. Candidate biomarkers did not show high correlations. Shades of red indicate a positive correlation coefficient, white indicates a correlation coefficient of zero, and shades of blue indicate a negative correlation coefficient. The correlation coefficient for each pairwise comparison is reported in the corresponding box. Only 12 candidate biomarkers are shown because five markers are categorical variables with relatively few categories.
