# Supplementary material for: An Alzheimer’s Disease-Derived Biomarker Signature Identifies Parkinson’s Disease Patients with Dementia
Source: PLoS One. 2016 Jan 26;11(1):e0147319. doi: 10.1371/journal.pone.0147319 (PMC4727929; doi:10.1371/journal.pone.0147319)
Supplement: S1 Methods — (DOCX) [file pone.0147319.s004.docx]

**S1 Methods**

**Participants**

**Penn Udall Cohort:**

Patients in the present cohort (n = 75) met the UK Brain Bank diagnostic criteria for PD [1]. Plasma, CSF, DNA, clinical data, demographic information, neuropsychological testing, and magnetic resonance imaging (MRI) were obtained from all patients as detailed in following sections. These individuals represent the first 75 subjects prospectively enrolled for the Intensive Assessment Cohort (IAC) of the UPenn Udall Center, which has a planned enrollment of 150 PD patients.

For each patient, all candidate biomarkers were captured within one year of the baseline visit. Specifically, genetic, biochemical, and imaging data were used from the closest available date within one year of the research visit date. The research visit date is the date at which clinical and neuropsychological measures were obtained.

Candidate biomarkers, along with literature supporting their nomination, are summarized in **S1 Table**. Candidate biomarkers were nominated from the existing literature on cognitive biomarkers in PD or AD. In most cases, each biomarker had only been previously studied as an individual predictor of cognitive status, with or without adjustment for demographic variables.

Informed consent was obtained from patients or their authorized representatives (in the case of individuals with limitations on capacity to consent) under IRB approval from the University of Pennsylvania. We note that investigators affiliated with our center have conducted research on the specific question of research consent capacity in PD for years (R01NS065087, PI J. Karlawish; NINDS), and the clinical core of the Penn Udall Center (which recruits patients) has worked under their guidance with respect to the issue of capacity to consent.

**ADNI Cohort:**

Data used in the preparation of this article were obtained from the Alzheimer’s Disease Neuroimaging Initiative (ADNI) database (adni.loni.usc.edu). The ADNI was launched in 2003 by the National Institute on Aging (NIA), the National Institute of Biomedical Imaging and Bioengineering (NIBIB), the Food and Drug Administration (FDA), private pharmaceutical companies and non-profit organizations, as a $60 million, 5-year public-private partnership. The primary goal of ADNI has been to test whether serial magnetic resonance imaging (MRI), positron emission tomography (PET), other biological markers, and clinical a neuropsychological assessment can be combined to measure the progression of mild cognitive impairment (MCI) and early Alzheimer’s disease (AD). Determination of sensitive and specific markers of very early AD progression is intended to aid researchers and clinicians to develop new treatments and monitor their effectiveness, as well as lessen the time and cost of clinical trials.

The Principal Investigator of this initiative is Michael W. Weiner, MD, VA Medical Center and University of California – San Francisco. ADNI is the result of efforts of many co-investigators from a broad range of academic institutions and private corporations, and subjects have been recruited from over 50 sites across the U.S. and Canada. The initial goal of ADNI was to recruit 800 subjects but ADNI has been followed by ADNI-GO and ADNI-2. To date these three protocols have recruited over 1500 adults, ages 55 to 90, to participate in the research, consisting of cognitively normal older individuals, people with early or late MCI, and people with early AD. The follow up duration of each group is specified in the protocols for ADNI-1, ADNI-2 and ADNI-GO. Subjects originally recruited for ADNI-1 and ADNI-GO had the option to be followed in ADNI-2. For up-to-date information, see www.adni-info.org.

Specifically, for this project, CSF Aβ42, CSF t-tau, CSF p-tau, SPARE-AD score, and *APOE* genotype were downloaded from the ADNI website for all cognitively normal (n=109) and AD (n=101) patients with complete information available for all five biomarkers. The only exclusion criterion applied was incomplete data.

**Classification of PD patients as PD-CN, PD-MCI, or PDD**

Cognitive status (cognitively normal (PD-CN), mild cognitive impairment (PD-MCI), or dementia (PDD)) was determined by expert clinical consensus at the UPenn Udall Center as previously described [2]. Assignment of a cognitive diagnosis is made for each patient at baseline and at every follow-up visit during a consensus conference held every six months by movement disorders specialists affiliated with the UPenn Udall Center. The consensus process involves multiple (five on average) pairs of experienced physician raters reviewing demographic and clinical data (including the clinician or patient impression of cognitive decline compared with premorbid state, multiple measures of function in activities of daily living, and psychometric test data). The physician raters assign patients a diagnosis of normal cognition, MCI, or dementia for each visit based on these data and following the diagnostic criteria proposed by the MDS Task Forces for MCI (Level 1 criteria from 2010-2014, Level II criteria from 2015 onward) and dementia [3,4]. Raters are not blinded to previous years’ cognitive diagnoses for a given patient. First, the raters within a pair reach agreement on all cases assigned to them. Between-pair inter-rater agreement over time was assessed in 137 cases assigned to two pairs. Agreement between pairs of raters was high (kappa = 0.80, 95% confidence interval = 0.70-0.90). For cases with a between-pair discrepancy in diagnosis, an independent physician rater adjudicates.

**Motor Assessment**

Motor severity of PD symptoms was assessed by the Unified Parkinson's Disease Rating Scale Part III (UPDRS-III) score [5] and by the Modified Hoehn and Yahr (MODHY) score [6,7].

**Genetic Testing**

***APOE*, *MAPT*, and *COMT***

DNA was extracted from peripheral blood using either the Flexigene Kit (Qiagen) or QuickGene DNA whole blood kit L (Autogen) and genotyped using real-time allelic discrimination with Applied Biosystem (ABI) TaqMan probes as previously described [8]. Primers were designed to genotype the following SNPs: *MAPT* (rs1052553, C_7563736_10), COMT p.V158M (rs4680, C_25746809_50), and *APOE* (rs7412, C_904973_10 and rs429358, C_3084793_20).

***GBA***

Genotyping was performed as previously described using long-range polymerase chain reaction amplification [9]. All 11 *GBA* exons and intron-exon boundaries were sequenced using the Applied Biosystems Big-Dye Terminator v3.1 Cycle Sequencing Kit on an ABI PRISM 3130 genetic analyzer (Applied Biosystems, Foster City, CA). Sequence data were base-called, aligned, and scanned for variation using Mutation Surveyor (SoftGenetics, State College, PA).

**Biochemical Testing**

CSF biomarkers were measured as previously described [10,11]. In brief, CSF was collected by lumbar puncture at the ADNI sites and the UPenn Medical Center and stored at -80°C. Aβ42 , t-tau, and p-tau were measured with Innogenetics (INNOBIA AlzBio3; Ghent, Belgium; for research-use only reagents) immunoassay kit-based reagent on the xMAP Luminex platform as previously described [10,11].

Whole blood samples were collected at UPenn in 10 mL K_2_EDTA tubes, immediately placed on ice, and spun down at 3,000 rpm for 5 minutes at 4°C within 24 hours. Plasma samples were stored at -80°C until use. EGF concentrations were measured as previously described [12] on a commercially available enzyme-linked immunosorbent assay (ELISA, R&D Systems, Minneapolis, MN). Samples were run in duplicate and EGF values were included in the analyses if they passed our quality control (QC) measure by having a coefficient of variation less than 0.2. Ninety-five percent (71/75) of samples passed this QC measure and were included in our analyses.

**Imaging**

A score for the Spatial Pattern of Atrophy for Recognition of Alzheimer’s Disease (SPARE-AD) was assigned for each participant as previously described [13]. In brief, the SPARE-AD score reflects the overall similarity between pattern of atrophy seen in a particular individual and a generic pattern reflective of AD.

**Statistical Analysis**

All statistical analyses were performed in R (http://www.r-project.org). Scripts are available in **S1 Scripts**.

**Multiple Imputation**

A panel of 17 biomarkers was assessed in all patients for a 98% complete dataset (1251/1275 data points available). The 24 missing data points were multiply imputed using the “mi” package in R [14]. Each biomarker with missing values had these values imputed by 30 iterative regression imputations using a bootstrap method until approximate convergence. For details, see “mi” package description at <http://cran.r-project.org/web/packages/mi/mi.pdf>.

**Bivariate Analyses**

Comparisons of PD-CN vs. PDD for the eight candidate biomarkers for which a cross-sectional effect had been previously reported (SPARE-AD, plasma EGF, CSF p-tau, CSF t-tau, CSF Aβ42 , GDS, MODHY, and UPDRS III) were evaluated by the non-parametric Mann-Whitney U-test. Bonferroni correction was used to adjust for multiple comparisons.

**Correlation Analyses**

Ordinalization of genotype data was performed; ordinalization was binary for *MAPT* (denoting H1/H1 haplotype vs. other haplotype) and *GBA* (denoting presence/absence of *GBA* mutation). For *APOE* and *COMT*, values were 0,1, or 2, indicating the number of E4 alleles or Met alleles, respectively. Pairwise Spearman correlation coefficients were calculated for assessment of internal correlations among

continuous markers and markers with at least 5 ranked categories. Partial pairwise Spearman correlation coefficients, adjusted for cognition (age-adjusted DRS score) were calculated in a similar manner.

To examine correlation between two categorical variables, logistic regression was used and R^2^ (McFadden) reported. To examine correlation between one categorical and one continuous variable, linear regression was used and R^2^ reported. Prior to linear regression, distributions of biomarkers were tested for normality (Shapiro-Wilk test); biomarkers that were non-normally distributed were log-transformed (CSF t-tau, age, disease duration, plasma EGF).

**Hierarchical Clustering, and Logistic Regression Classifier**

Patients and biomarkers were hierarchically clustered by Euclidean distance using average linkage, and heatmaps were generated for visualization of internal relationships. Prior to clustering, distributions for biomarkers were tested for normality (Shapiro-Wilk test); biomarkers that were non-normally distributed were log-transformed (CSF t-tau, age, disease duration, plasma EGF), then standardized by setting the mean of each variable to zero with a standard deviation of one.

For classification of AD vs. cognitively normal samples, a five-marker logistic regression classifier was trained on ADNI data and ten-fold cross-validated. The classifier was then evaluated for its ability to identify PDD patients in the Penn Udall cohort. 95% confidence intervals were obtained using the Clopper-Pearson method.

**References**

1. Gibb WR, Lees AJ. The relevance of the Lewy body to the pathogenesis of idiopathic Parkinson's disease. J Neurol Neurosurg Psychiatry. 1988;51: 745-752.

2. Chahine LM, Qiang J, Ashbridge E, Minger J, Yearout D, Horn S, et al. Clinical and biochemical differences in patients having Parkinson disease with vs without GBA mutations. . 2013;70: 852-858.

3. Litvan I, Goldman JG, Troster AI, Schmand BA, Weintraub D, Petersen RC, et al. Diagnostic criteria for mild cognitive impairment in Parkinson's disease: Movement Disorder Society Task Force guidelines. Mov Disord. 2012;27: 349-356.

4. Emre M, Aarsland D, Brown R, Burn DJ, Duyckaerts C, Mizuno Y, et al. Clinical diagnostic criteria for dementia associated with Parkinson's disease. Mov Disord. 2007;22: 1689-707; quiz 1837.

5. Fahn S, Elton R. Unified rating scale for Parkinson’s disease. . 1987: 153-163.

6. Hoehn M. Yahr. . 1967: V7.

7. Jankovic J, McDermott M, Carter J, Gauthier S, Goetz C, Golbe L, et al. Variable expression of Parkinson's disease: a base-line analysis of the DATATOP cohort. The Parkinson Study Group. Neurology. 1990;40: 1529-1534.

8. Morley JF, Xie SX, Hurtig HI, Stern MB, Colcher A, Horn S, et al. Genetic influences on cognitive decline in Parkinson's disease. . 2012;27: 512-518.

9. Tsuang D, Leverenz JB, Lopez OL, Hamilton RL, Bennett DA, Schneider JA, et al. GBA mutations increase risk for Lewy body disease with and without Alzheimer disease pathology. Neurology. 2012;79: 1944-1950.

10. Shaw LM, Vanderstichele H, Knapik‐Czajka M, Clark CM, Aisen PS, Petersen RC, et al. Cerebrospinal fluid biomarker signature in Alzheimer's disease neuroimaging initiative subjects. Ann Neurol. 2009;65: 403-413.

11. Olsson A, Vanderstichele H, Andreasen N, De Meyer G, Wallin A, Holmberg B, et al. Simultaneous measurement of beta-amyloid(1-42), total tau, and phosphorylated tau (Thr181) in cerebrospinal fluid by the xMAP technology. Clin Chem. 2005;51: 336-345.

12. Chen‐Plotkin AS, Hu WT, Siderowf A, Weintraub D, Goldmann Gross R, Hurtig HI, et al. Plasma epidermal growth factor levels predict cognitive decline in Parkinson disease. Ann Neurol. 2011;69: 655-663.

13. Weintraub D, Dietz N, Duda JE, Wolk DA, Doshi J, Xie SX, et al. Alzheimer's disease pattern of brain atrophy predicts cognitive decline in Parkinson's disease. Brain. 2012;135: 170-180.

14. Su Y, Yajima M, Gelman AE, Hill J. Multiple imputation with diagnostics (mi) in R: Opening windows into the black box. . 2011;45: 1-31.
