# Supplementary material for: An Alzheimer’s Disease-Derived Biomarker Signature Identifies Parkinson’s Disease Patients with Dementia
Source: PLoS One. 2016 Jan 26;11(1):e0147319. doi: 10.1371/journal.pone.0147319 (PMC4727929; doi:10.1371/journal.pone.0147319)
Supplement: S1 Scripts — (DOCX) [file pone.0147319.s005.docx]

**S1 Scripts**

##############Correlations – Partial Correlations

install.packages("ppcor")

install.packages("corrplot")

library(ppcor)

library(corrplot)

col1 <- colorRampPalette(c("blue","white", "red"))

###Set working directory here: setwd(...)

imputed=read.csv("CorrelationData.csv", check.names=FALSE)

str(imputed)

#Initialize partial correlation matrix

pcorrmat<-matrix(1,12,12)

rownames(pcorrmat)=colnames(imputed)[2:13]

colnames(pcorrmat)=colnames(imputed)[2:13]

#Loop through all non-INDDID/DRSTotalAge columns

for (i in 2:13){

#Pairwise loop through all other predictors and calculate partial correlation, controlled for DRSTotalAge

if(i<=12){for (j in (i+1):13){

temp<-pcor.test(imputed[[i]],imputed[[j]],imputed[[14]], method="spearman")$estimate

pcorrmat[i-1,j-1]<-temp

pcorrmat[j-1,i-1]<-temp

#cat(i,",",j,"\n")

}}

}

###Create correlograms

#With superimposed correlation coefficients

dev.new()

corrplot(pcorrmat, type="lower", method="shade", col = col1(100),

addCoef.col="black",

tl.col= NULL, tl.srt=45, tl.cex=0.7)

#Without superimposed correlation coefficients

dev.new()

corrplot(pcorrmat, type="lower", method="shade", col = col1(100),

addCoef.col=NULL,

tl.col= NULL, tl.srt=45, tl.cex=0.7)

#Mixed-style full correlogram

dev.new()

corrplot(pcorrmat, type="lower", method="shade", col = col1(100),

addCoef.col="black", diag=T,

tl.pos="lt", tl.col= NULL, tl.srt=45, tl.cex=0.7)

corrplot(pcorrmat, type="upper", add=T, method="shade", col = col1(100),

addCoef.col=NULL, diag=F,

tl.pos="n", tl.col= NULL, tl.srt=45, tl.cex=0.7)

#######Correlations – Spearman

install.packages("corrplot")

library(corrplot)

col1 <- colorRampPalette(c("blue","white", "red"))

## Set working directory here

imputed<-read.csv("CorrelationData.csv", check.names=FALSE)

trimmed<-imputed[,-c(1,14,15)]

str(trimmed)

corr<-cor(trimmed, method="spearman")

#Create correlogram with superimposed correlation coefficients

dev.new()

corrplot(corr, type="lower", method="shade", col = col1(100),

addCoef.col="black",

tl.col= NULL, tl.srt=45, tl.cex=0.7)

######################Logistic Regression Classifier Script

####Install and read in necessary packages

install.packages("ROCR")

install.packages("pROC")

install.packages("caret")

library("pROC")

library("ROCR")

library("caret")

#Read in datasheets

ADNI=read.csv("ADNI Patients AD and CN.csv", check.names=FALSE)

Udall_CN_D=read.csv("D N with AD biomarkers only.csv", check.names=FALSE)

#Remove IDs to leave working data

ADNI<-ADNI[,-1]

Udall_CN_D<-Udall_CN_D[,-1]

#Log transform CSF t-tau

ADNI[,3] = log(ADNI[,3])

Udall_CN_D[,3] = log(Udall_CN_D[,3])

#Normalize data to a mean of 0 and standard deviation of 1

ADNI.normal=scale(ADNI[1:5], scale=TRUE, center=TRUE)

Udall_CN_D.normal=scale(Udall_CN_D[1:5], scale=TRUE, center=TRUE)

#Create datasheet that is normalized and has true outcome label (i.e. PDD,PD-CN, etc.)

ADNI_ord1=data.frame(ADNI.normal[,1:5], ADNI[7])

Udall_CN_D_ord=data.frame(Udall_CN_D.normal[,1:5], Udall_CN_D[7])

############Create logistic regression classifier

set.seed(1050)

ctrl <- trainControl(method = "cv", number=10, classProbs = TRUE, summaryFunction =twoClassSummary)

plsFit=train(x=ADNI_ord1[1:5],y=ADNI_ord1$Diagnosis,method="glm",trControl = ctrl, metric="ROC")

set.seed(1050)

ctrl2 <- trainControl(method = "cv", number=10, classProbs = TRUE, summaryFunction =defaultSummary)

plsFit2=train(x=ADNI_ord1[1:5],y=ADNI_ord1$Diagnosis,method="glm",trControl = ctrl2)

specificity=as.numeric(plsFit[[4]][4])

sensitivity=as.numeric(plsFit[[4]][3])

accuracy=as.numeric(plsFit2[[4]][2])

AUC=as.numeric(plsFit[[4]][2])

#store performance stats of logistic classifer

GLM_stats=matrix(nrow=10,ncol=4)

colnames(GLM_stats)=c("AUC","Accuracy","Sensitivity","Specificity")

#store probs and labels of classifiers needed for ROC analysis

GLM.probs=vector()

GLM.labels=vector()

#do 10-fold cross validation for trainset as a check- allows plotting of ROC curve for training set

# plsFit$control$index[[i]]: indicies of the 9 folds

# plsFit$control$indexOut[[i]]: 1 left out fold

#iterate from i=1 to 10 to capture all 10-fold combinations

for(i in 1:10){

ctrl <- trainControl(method = "cv", number=10, classProbs = TRUE, summaryFunction =twoClassSummary)

Logistic_model=train(x=ADNI_ord1[1:5],y=ADNI_ord1$Diagnosis,method="glm",trControl = ctrl, metric="ROC")

GLM_Classes <- predict(Logistic_model, newdata = ADNI_ord1[plsFit$control$indexOut[[i]],-6])

GLM_probs <- predict(Logistic_model, newdata = ADNI_ord1[plsFit$control$indexOut[[i]],-6], type = "prob")$AD

matrix=confusionMatrix(data = GLM_Classes, ADNI_ord1[plsFit$control$indexOut[[i]],6])

GLM_prediction <- predict(Logistic_model, ADNI_ord1[plsFit$control$indexOut[[i]],-6])

GLM_sensitivity=matrix[[4]][2]

GLM_specificity=matrix[[4]][1]

GLM_accuracy=matrix[[3]][1]

GLM_lab=ADNI_ord1[plsFit$control$indexOut[[i]],6]== "AD"

GLM.rocr=prediction(GLM_probs, GLM_lab)

GLM.auc=performance(GLM.rocr,"auc")

GLM_AUC=attributes(GLM.auc)$y.values[[1]]

GLM_stats[i,]=c(GLM_AUC, GLM_accuracy, GLM_sensitivity, GLM_specificity)

GLM.probs=c(GLM.probs,GLM_probs)

GLM.labels=c(GLM.labels,GLM_lab)

}

###Store performance data

GLM.rocr=prediction(as.vector(GLM.probs), as.vector(GLM.labels))

GLM.perf<-performance(GLM.rocr, measure = "tpr", x.measure = "fpr")

##########Test classifier on separate test cohort (Udall) predicting demented vs. normal

plsClasses <- predict(plsFit, newdata = Udall_CN_D_ord[1:5])

plsProbs <- predict(plsFit, newdata = Udall_CN_D_ord[1:5], type="prob")$AD

pls.label=Udall_CN_D_ord$consensusDX=="Dementia"

pls.rocr=prediction(plsProbs, pls.label)

pls.auc=performance(pls.rocr, "auc")

pls.acc=performance(pls.rocr, "acc")

pls.plot= performance(pls.rocr, measure = "tpr", x.measure = "fpr")

plot(pls.plot)

perf=performance(pls.rocr, "sens", "spec")

####Performance metrics

RAUC=attributes(pls.auc)$y.values[[1]]

spec =attributes(perf)$x.values[[1]]

sens = attributes(perf)$y.values[[1]]

acc = attributes(pls.acc)$y.values[[1]]

####Plot ADNI and Udall data on the same graph

plot.new()

dev.new()

pdf(file="Logistic Classifer.pdf", height=12.54/1.3, width=12/1.3)

plot(pls.plot)

plot(pls.plot,col="red", add=TRUE,lwd=3)

plot(GLM.perf,col=1, add=TRUE,lwd=3)

box(lwd=3)

abline(coef=c(0,1),lty=2)

legend("bottomright",legend=c("Training Cohort Normal vs. AD", "AUC: 0.992", "Accuracy: 0.963", "Sens: 0.961, Spec: 0.964", "5 Biomarker Panel PD-CN vs. PDD","AUC: 0.867","Accuracy: 0.800","Sens: 0.875, Spec: 0.787"),lty=c(1,0,0,0,1,0,0,0),col=c(1,1,1,"red","red","red"),text.col=c(1,1,1,1,"red","red","red","red"),lwd=c(2,0,0,0,2,0,0,0))

title("Logistic Classifer Performance")

dev.off()

###################For supplement: Calculation of performance metrics without imputed datapoint

Udall_no_102975= rbind(Udall_CN_D_ord[1:5,],Udall_CN_D_ord[7:55,])

plsClasses <- predict(plsFit, newdata = Udall_no_102975[,1:5] )

plsProbs <- predict(plsFit, newdata = Udall_no_102975[,1:5], type="prob")$AD

pls.label=Udall_no_102975$consensusDX=="Dementia"

pls.rocr=prediction(plsProbs, pls.label)

pls.auc=performance(pls.rocr, "auc")

pls.acc=performance(pls.rocr, "acc")

pls.plot= performance(pls.rocr, measure = "tpr", x.measure = "fpr")

plot(pls.plot)

perf=performance(pls.rocr, "sens", "spec")

####Performance metrics

RAUC2=attributes(pls.auc)$y.values[[1]]

spec2 =attributes(perf)$x.values[[1]]

sens2 = attributes(perf)$y.values[[1]]

acc2 = attributes(pls.acc)$y.values[[1]]
