# Supplementary material for: An Alzheimer’s Disease-Derived Biomarker Signature Identifies Parkinson’s Disease Patients with Dementia
Source: PLoS One. 2016 Jan 26;11(1):e0147319. doi: 10.1371/journal.pone.0147319 (PMC4727929; doi:10.1371/journal.pone.0147319)
Supplement: S1 Table — 16 markers previously reported in the literature are summarized, but 17 were evaluated in the present study, since motor severity was assessed by both UPDRS-III and MODHY. Full references are provided in the main text. (DOCX) [file pone.0147319.s006.docx]

|  | **Marker** | **Direction** | **Reference** |
| --- | --- | --- | --- |
| **Clinical** | Age | Age ≥ 72 associated with more rapid decline in Mini-Mental State Exam longitudinally | **4.**  Williams-Gray CH et al. Brain. 2007 |
|  | Motor Severity | UPDRS-III ≥ 25 associated with greater risk of dementia at baseline and longitudinally | **15.**  Marder KM et al.  Arch Neurol. 1995 |
|  | Sex | 2.95 times greater risk with male sex | **16.**  UC et al.  Neurology. 2009 |
|  | Motor phenotype | 4.1 times greater risk of dementia with mixed/PIGD motor phenotype | **4.**  Williams-Gray CH et al. Brain. 2007 |
|  | Disease duration | Longer disease duration may be associated with greater risk | **17.**  Hughes et al. Neurology. 2000 |
|  | Hallucinations | UPDRS Thought Disorder ≥ 1 associated with greater risk of dementia longitudinally | **18.**  Aarsland D et al. Neurology. 2001 |
|  | Depression | Hamilton Depression Rating Scale > 10 associated with greater risk of dementia at baseline and longitudinally | **15.**  Marder KM et al.  Arch Neurol. 1995 |
| **Genetic** | *APOE* | 2.8 times risk of ≥ 10-point drop in DRS-2 for E4 carriers | **19.**  Morley JF et al.  Mov Disord. 2012 |
|  | *MAPT* | 12 times greater risk for conversion to PD longitudinally for H1/H1 haplotype | **20.**  Williams-Gray CH et al. Brain. 2009 |
|  | *COMT* | Greater executive dysfunction as measured by Tower of London with each met allele | **20.**  Williams-Gray CH et al. Brain. 2009 |
|  | *GBA* | 48% of mutation carriers experienced cognitive decline or dementia | **21.**  Neumann J et al.  Brain. 2009 |
| **Biochemical** | CSF Aβ42 | Lower baseline CSF AB1-42 associated with more rapid cognitive decline | **22.**  Siderowf A et al. Neurology. 2010 |
|  | CSF t-tau | Increased in AD | **23.**  Shaw L et al.  Ann. Neurol. 2009 |
|  | CSF p-tau | Increased in AD | **23.**  Shaw L et al.  Ann. Neurol. 2009 |
|  | Plasma EGF | 8.34 times greater risk of dementia with lowest quartile of plasma EGF longitudinally. Lower EGF associated with worse baseline cognition. | **25.**  Chen-Plotkin A et al. Ann. Neurol. 2011 |
| **Imaging** | SPARE-AD | Higher baseline SPARE-AD score associated with cognitive decline longitudinally and worse cognitive performance at baseline. | **28.**  Weintraub D et al. Brain. 2011 |

**S1 Table. Previously-reported associations between candidate biomarkers and cognition**. 16 markers previously reported in the literature are summarized, but 17 were evaluated in the present study, since motor severity was assessed by both UPDRS-III and MODHY. Full references are provided in the main text.
