# Supplementary material for: An Alzheimer’s Disease-Derived Biomarker Signature Identifies Parkinson’s Disease Patients with Dementia
Source: PLoS One. 2016 Jan 26;11(1):e0147319. doi: 10.1371/journal.pone.0147319 (PMC4727929; doi:10.1371/journal.pone.0147319)
Supplement: S2 Table — We assessed the degree of internal correlation among our candidate markers by calculating R2 values for logistic and linear regressions involving one or more categorical markers. For categorical-categorical biomarker comparisons (shaded in grey), logistic regressions were performed and the McFadden R2 value is reported. For categorical-continous biomarker comparisons, linear regressions were performed after inspection of data for normality, and log-transformation of non-normal biomarkers. The degree of correlation observed among markers was low. (DOCX) [file pone.0147319.s007.docx]

|  | **Sex** | ***APOE*** | ***COMT*** | ***GBA*** | ***MAPT*** |
| --- | --- | --- | --- | --- | --- |
| **Sex** | 1.00 | 0.00 | 0.04 | 0.01 | 0.01 |
| ***APOE*** | 0.00 | 1.00 | 0.03 | 0.05 | 0.04 |
| ***COMT*** | 0.00 | 0.02 | 1.00 | 0.02 | 0.00 |
| ***GBA*** | 0.04 | 0.26 | 0.17 | 1.00 | 0.07 |
| ***MAPT*** | 0.01 | 0.05 | 0.01 | 0.02 | 1.00 |
| **Age** | 0.00 | 0.00 | 0.15 | 0.01 | 0.04 |
| **MODHY** | 0.01 | 0.00 | 0.03 | 0.00 | 0.01 |
| **UPDRS III** | 0.05 | 0.01 | 0.05 | 0.00 | 0.01 |
| **Tremor:PIGD Ratio** | 0.02 | 0.00 | 0.03 | 0.02 | 0.00 |
| **Disease Duration** | 0.00 | 0.00 | 0.06 | 0.00 | 0.00 |
| **Hallucinations** | 0.00 | 0.07 | 0.04 | 0.00 | 0.01 |
| **GDS** | 0.01 | 0.07 | 0.01 | 0.02 | 0.00 |
| **CSF AB42** | 0.03 | 0.10 | 0.01 | 0.03 | 0.00 |
| **CSF T-Tau** | 0.02 | 0.01 | 0.06 | 0.08 | 0.00 |
| **CSF P-Tau** | 0.00 | 0.04 | 0.01 | 0.00 | 0.00 |
| **Plasma EGF** | 0.00 | 0.00 | 0.03 | 0.02 | 0.01 |
| **SPARE-AD** | 0.04 | 0.02 | 0.00 | 0.00 | 0.06 |

**S2 Table.** **R^2^ values for correlation of categorical markers.**

We assessed the degree of internal correlation among our candidate markers by calculating R^2^ values for logistic and linear regressions involving one or more categorical markers. For categorical-categorical biomarker comparisons (shaded in grey), logistic regressions were performed and the McFadden R^2^ value is reported. For categorical-continous biomarker comparisons, linear regressions were performed after inspection of data for normality, and log-transformation of non-normal biomarkers. The degree of correlation observed among markers was low.
