# Supplementary material for: An Alzheimer’s Disease-Derived Biomarker Signature Identifies Parkinson’s Disease Patients with Dementia
Source: PLoS One. 2016 Jan 26;11(1):e0147319. doi: 10.1371/journal.pone.0147319 (PMC4727929; doi:10.1371/journal.pone.0147319)
Supplement: S3 Table — Logistic regression models predicting cognitive category (PDD vs. PD-CN) based on indicated variables. Five models are shown. In each case, the addition of the biochemical or imaging biomarker improves the model compared to age alone, as reflected by a lower Akaike Information Criterion (AIC) measure. In each case, the addition of age as a covariate does not change the direction and minimally changes the magnitude of effect for the biochemical or imaging biomarker, as reflected by the coefficient. (DOCX) [file pone.0147319.s008.docx]

| Model | Variables | Coefficient | P-Value | AIC |
| --- | --- | --- | --- | --- |
| 1 | Age | -0.26 | 0.003 | 34.10 |
| 2 | SPARE-AD | -1.31 | 0.016 | 40.93 |
| 3 | SPARE-AD  Age | -1.26  -0.26 | 0.040  0.008 | 30.12 |
| 4 | CSF Aβ42 | 0.02 | 0.005 | 39.32 |
| 5 | CSF Aβ42  Age | 0.01  -0.21 | 0.088  0.019 | 32.81 |

**S3 Table. Effect of age on classifier.**

Logistic regression models predicting cognitive category (PDD vs. PD-CN) based on indicated variables. Five models are shown. In each case, the addition of the biochemical or imaging biomarker improves the model compared to age alone, as reflected by a lower Akaike Information Criterion (AIC) measure. In each case, the addition of age as a covariate does not change the direction and minimally changes the magnitude of effect for the biochemical or imaging biomarker, as reflected by the coefficient.
